# The Origins of Lactase Persistence in Europe

**DOI:** 10.1371/journal.pcbi.1000491

**Published:** 2009-08-28

**Authors:** Yuval Itan, Adam Powell, Mark A. Beaumont, Joachim Burger, Mark G. Thomas

**Affiliations:** 1 Research Department of Genetics, Evolution and Environment, University College London, London, United Kingdom; 2 School of Animal and Microbial Sciences, The University of Reading, Whiteknights, Reading, United Kingdom; 3 Johannes Gutenberg University, Institute of Anthropology, Mainz, Germany; 4 CoMPLEX (Centre for Mathematics & Physics in the Life Sciences and Experimental Biology), University College London, London, United Kingdom; 5 AHRC Centre for the Evolution of Cultural Diversity, Institute of Archaeology, University College London, London, United Kingdom; University of New South Wales, Australia

## Abstract

Lactase persistence (LP) is common among people of European ancestry, but with the exception of some African, Middle Eastern and southern Asian groups, is rare or absent elsewhere in the world. Lactase gene haplotype conservation around a polymorphism strongly associated with LP in Europeans (*−13,910 C/T*) indicates that the derived allele is recent in origin and has been subject to strong positive selection. Furthermore, ancient DNA work has shown that the −13,910*T (derived) allele was very rare or absent in early Neolithic central Europeans. It is unlikely that LP would provide a selective advantage without a supply of fresh milk, and this has lead to a gene-culture coevolutionary model where lactase persistence is only favoured in cultures practicing dairying, and dairying is more favoured in lactase persistent populations. We have developed a flexible demic computer simulation model to explore the spread of lactase persistence, dairying, other subsistence practices and unlinked genetic markers in Europe and western Asia's geographic space. Using data on −13,910*T allele frequency and farming arrival dates across Europe, and approximate Bayesian computation to estimate parameters of interest, we infer that the −13,910*T allele first underwent selection among dairying farmers around 7,500 years ago in a region between the central Balkans and central Europe, possibly in association with the dissemination of the Neolithic Linearbandkeramik culture over Central Europe. Furthermore, our results suggest that natural selection favouring a lactase persistence allele was not higher in northern latitudes through an increased requirement for dietary vitamin D. Our results provide a coherent and spatially explicit picture of the coevolution of lactase persistence and dairying in Europe.

## Author Summary

Most adults worldwide do not produce the enzyme lactase and so are unable to digest the milk sugar lactose. However, most people in Europe and many from other populations continue to produce lactase throughout their life (lactase persistence). In Europe, a single genetic variant, −13,910*T, is strongly associated with lactase persistence and appears to have been favoured by natural selection in the last 10,000 years. Since adult consumption of fresh milk was only possible after the domestication of animals, it is likely that lactase persistence coevolved with the cultural practice of dairying, although it is not known when lactase persistence first arose in Europe or what factors drove its rapid spread. To address these questions, we have developed a simulation model of the spread of lactase persistence, dairying, and farmers in Europe, and have integrated genetic and archaeological data using newly developed statistical approaches. We infer that lactase persistence/dairying coevolution began around 7,500 years ago between the central Balkans and central Europe, probably among people of the Linearbandkeramik culture. We also find that lactase persistence was not more favoured in northern latitudes through an increased requirement for dietary vitamin D. Our results illustrate the possibility of integrating genetic and archaeological data to address important questions on human evolution.

## Introduction

Lactase persistence (LP) is an autosomal dominant trait enabling the continued production of the enzyme lactase throughout adult life. Lactase non-persistence is the ancestral condition for humans, and indeed for all mammals [1]. Production of lactase in the gut is essential for the digestion of the milk sugar lactose. LP is common in northern and western Europeans as well as in many African, Middle Eastern and southern Asian pastoralist groups, but is rare or absent elsewhere in the world [1]–[4]. In Europeans LP is strongly associated with a single C to T transition in the *MCM6* gene (−13,910*T), located 13.91 kb upstream from the lactase gene [5]. Furthermore, *in vitro* studies have indicated that the −13,910*T allele can directly affect *LCT* gene promoter activity [6]. The −13,910*T allele ranges frequency from 6%–36% in eastern and southern Europe, 56%–67% in Central and western Europe, to 73%–95% in the British Isles and Scandinavia [7],[8] while LP ranges in frequency from 15%–54% in eastern and southern Europe, 62%–86% in Central and western Europe, to 89%–96% in the British Isles and Scandinavia [9]. This makes the −13,910*T allele a good candidate for predicting LP in Europe. However, genotype/phenotype frequency comparisons have shown that the −13,910*T allele cannot account for LP frequencies in most African [3] and Middle Eastern populations [10]. Instead, different LP-associated alleles occurring in the same genomic region have been reported, indicating convergent evolution [2],[4],[10],[11].

Using long-range haplotype conservation [8] and variation in closely linked microsatellites [12] as proxies for allelic age, the −13,910*T variant has been estimated to be between 2,188 and 20,650 years old and between 7,450 and 12,300 years old, respectively. These recent age estimates, when considered in conjunction with modern allele frequencies, indicate that −13,910*T has been subjected to very strong natural selection (s = 0.014–0.19; [8]). It is interesting to note that similar estimates for the strength of selection have been obtained for one of the major African LP variants [4].

It is unlikely that lactase persistence would provide a selective advantage without a supply of fresh milk and this has lead to a gene-culture co-evolutionary model where lactase persistence is only favoured in cultures practicing dairying [13]–[16], and dairying is more favoured in lactase persistent populations [14], [17]–[19]. The reasons why LP, in conjunction with dairying, should confer such a strong selective advantage remain open to speculation. Flatz and Rotthauwe [20] proposed the *calcium assimilation hypothesis*, whereby a lactase persistence allele is favoured in high-latitude regions because reduced levels of sunlight do not allow sufficient synthesis of vitamin-D in the skin. Vitamin D is required for calcium absorption and milk provides a good dietary source of both nutrients. Additional factors are likely to include the ability to consume a calorie and protein-rich food source, the relative constancy in the supply of milk (in contrast to the boom-and-bust of seasonal crops), and the value of fresh milk as a source of uncontaminated fluids. It is likely that the relative advantages conferred by these various factors differ in Europe and Africa.

Estimates of the age of the −13,910*T correspond well with estimates of the onset of dairying in Europe. Slaughtering age profiles in sheep, goats and cattle suggest dairying was present in south-eastern Europe at the onset of the Neolithic [21],[22], while residual milk proteins preserved in ceramic vessels provide evidence for dairying in present day Romania and Hungary 7,900–7,450 years BP [23]. Furthermore, residual analyses of fats indicate dairying at the onset of the Neolithic in England, some 6,100 years BP [24],[25], and after to 8,500 BP in the western parts of present day Turkey [26]. Allelic age estimates are also consistent with the results of a recent ancient DNA study [27] which showed that the −13,910*T allele was rare or absent among early farmers from Central and Eastern Europe. These observations lend support to the view that −13,910*T, and thus LP, rose rapidly in frequency only after the onset of dairying, as opposed to the ‘reverse-cause’ hypothesis [14], [17]–[19], whereby dairying developed in response to the evolution of LP.

Important questions remain regarding the location of the earliest −13,910*T-carrying dairying groups and the demographic and gene-culture co-evolutionary processes that shaped the modern distribution of LP in Europe. The present-day distribution of the −13,910*T allele might be taken to indicate an origin in Northwest Europe. However, the earliest archaeozoological and residual lipid and protein evidence for dairying is found in the Near East, in Southeast Europe and in Mediterranean Europe [21],[26],[28]. While these observations can seem contradictory, forward computer simulations have shown that the centre of distribution of an allele can be far removed from its location of origin when a population expands along a wave front [29],[30].

Assuming that the −13,910*T-allele was only subjected to strong natural selection in dairying groups, it is likely that −13,910*T-carrying dairyers underwent demographic expansion to a greater extent than non-dairying groups. While gene flow between dairying and non-dairying groups would ultimately lead to genetic homogeneity, under conditions of limited gene flow between cultural groups, it is plausible that the earliest LP peoples would have made a higher contribution to the European gene pool than their non-LP neighbours. In this study we use demic forward computer simulations to examine potential scenarios for the spread of LP in Europe. We simulate three interacting cultural groups (hunter gatherers, non-dairying farmers and dairying farmers) and track the spread of an allele that is selected only in one group (dairying farmers). We also track the expected proportion of genetic ancestry from the geographic region where LP/dairying coevolution began. We parameterize intrademic gene flow between cultural groups, interdemic gene flow, sporadic longer-distance migration, the cultural diffusion of subsistence practices and selection favouring lactase persistent dairyers. We compare the predicted frequency of a LP allele and arrival dates of farmers – from simulation outcomes – to known frequencies of the −13,910*T allele [3],[8] and carbon-14 based estimates of the arrival dates of farmers [31] at different locations throughout Europe. We employ approximate Bayesian computation (ABC), a set of methods that allow the estimation of parameters under models too complex for a full-likelihood approach [32]. By comparing summary statistics on the observed data with those computed on our simulated datasets, ABC enables us to estimate the key demographic and evolutionary parameters including the region where LP-dairying coevolution in began in Europe.

## Results

### 

#### Simulation time

Unlike the simulation models used in related studies [33]–[36] the one we present is stochastic and more parameter-heavy. In addition, it was written in Python using the object orientated paradigm which, while utilizing some highly efficient array-handling libraries such as numarray and Numpy, is considerably slower than purely procedural simulations written in a lower-level programming language such as C++. A single simulation takes about 170 seconds on a 3.0 GHz Athlon™ 64 processor.

#### Demographic parameter estimation

**Figure 1:**
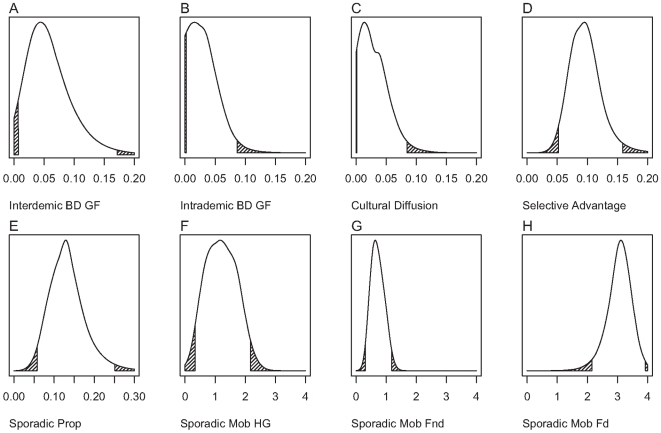
Approximate marginal posterior density estimates of demographic and evolutionary parameters. ABC was performed using regression adjustment and weighting, following acceptance at the 0.5% tolerance level [32]. The upper and lower 2.5% of each distribution are shaded. For some parameters the estimated 95% credible intervals lie outside the upper prior bound. This is a consequence of the regression adjustment stage of ABC when using rectangular priors [32]. Points in which the parameter value is close to the boundary, but with summary statistics that are distant from those observed, can have their parameter values projected outside the boundary. Parameters estimated are (A) Interdemic bidirectional geneflow, (B) Intrademic bidirectional geneflow, (C) the rate of cultural diffusion of subsistence practices, (D) the selective advantage of a LP allele among dairying farmers, (E) the proportion of individuals in a deme available for sporadic long-distance migration, and the average mobility – in number of demes moved – of (F) hunter-gatherers, (G) non-dairying farmers, and (H) dairying farmers.

We applied the regression adjustment and weighting step of ABC to simulations accepted at the 0.5% tolerance level [32]. As can be seen in Figure 1, for some parameters, such as the sporadic migration mobility of hunter-gatherers, little information could be obtained using the observed data (also see Table S2). This is unsurprising since we would expect the value for this parameter to make little difference to either the arrival time of farming or the distribution of a LP allele. However, our analyses did appear informative for some key parameters. (1) The 95% credibility interval (CI) for selective advantage of the LP allele among dairying farmers, *s*, is considerably narrower (0.0518–0.159; mode = 0.0953) than its prior (0–0.2); (2) The 95% CI for the proportion of individuals available for intrademic bidirectional geneflow between cultural groups, *P_c_*, (0.00206–0.0867; mode = 0.0153) falls in the lower end of its prior range (0–0.2); and (3) The sporadic migration mobility of dairying farmers, M_Fd_, is significantly higher than that for non-dairying farmers; 99.998% of 100,000 random draws from the former are greater that those from the latter. We note that for some parameters the estimated 95% credible intervals lie outside the upper prior bound. This is a consequence of using regression adjustment in a model with rectangular priors [32]. Points in which the parameter value is close to the boundary, but with summary statistics that are distant from those observed, may have their parameter values projected outside the boundary by the regression method.

**Figure 2:**
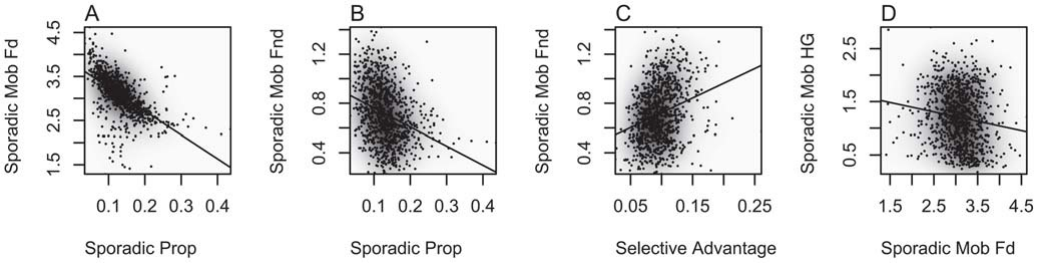
Pairwise joint approximate posterior density estimates of demographic and evolutionary parameters showing high degrees of correlation (Spearman's R^2^>0.024). Points represent regression adjusted parameter values from simulations accepted at the 0.5% tolerance level. Shading was added using 2D kernel density estimation. Parameter combinations shown are the proportion of individuals in a deme available for sporadic long-distance migration versus the average mobility – in number of demes moved – of (A) dairying farmers, and (B) non-dairying farmers, (C) the selective advantage of a LP allele among dairying farmers versus the average mobility of non-dairying farmers, and (D) the average mobility of dairying farmers versus the average mobility of hunter-gatherers.

To investigate relationships among demographic and evolutionary parameters we calculated Spearman's R^2^ and p-values for all possible pairwise joint posterior parameter distribution (see Supplementary Table S1), following acceptance at the 0.5% level and regression adjustment [32]. In Figure 2 we show those with R^2^>0.024. The following parameter pairs, in order of decreasing R^2^, showed non-independence by this criteria: (A) proportion available for sporadic migration and the sporadic mobility of dairying farmers, (B) proportion available for sporadic migration and the sporadic mobility of non-dairying farmers, (C) selective advantage and sporadic mobility of non-dairying farmers, and (D) sporadic mobility of dairying farmers and sporadic mobility of hunter-gatherers. That the first two joint distributions show negative correlation is unsurprising since changes in the proportion available for sporadic migration, or in the sporadic migration mobility of dairying and non-dairying farmers, will have similar effects on the timing of arrival of farming at different locations.

#### Geographic and temporal origin of LP-dairying co-evolution

**Figure 3:**
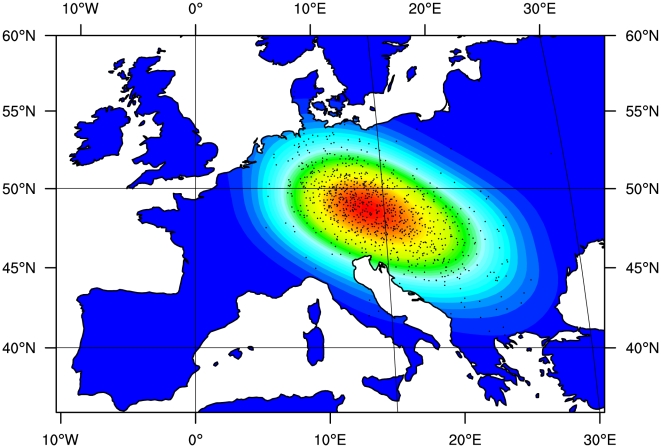
Approximate posterior density of region of origin for LP/dairying co-evolution. Points represent regression-adjusted latitude and longitude coordinates from simulations accepted at the 0.5% tolerance level. Shading was added using 2D kernel density estimation.

**Figure 4:**
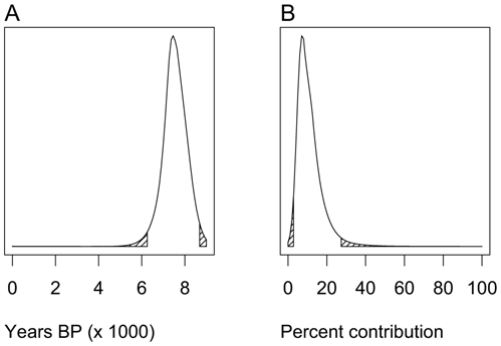
Estimates of the date of origin for LP/dairying coevolution and the contribution of people living in the deme of origin for LP/dairying co-evolution, and its eight surrounding demes, to the modern European gene pool. Although not parameters of the model *sensu stricto*, estimates were calculated as with all model parameters by using ABC with regression adjustment and weighting, following acceptance at the 0.5% tolerance level [32]. The date of origin for LP/dairying coevolution (A) is given in thousands of years before present, and the contribution of people living in the deme of origin for LP/dairying co-evolution, and its 8 surrounding demes, to the modern European gene pool (B) is given as a percentage. The upper and lower 2.5% of each distribution are shaded.

Following acceptance at the 0.5% level and regression adjustment we found that the most probable location where an LP allele first underwent selection among dairying farmers lies in a region between the central Balkans and central Europe (see Figure 3). It should be noted that, as simulated, we did not attempt to identify the location where the LP −13,910*T allele first arose. Instead we assumed that it started to rise to appreciable frequencies only after selection began among dairying farmers, initially at the particular location we estimated. The timing of the start of this gene-culture coevolution process was therefore strongly influenced by the arrival time of dairying farmers at the location where selection began in simulations. Since we selected simulations that give a good fit to the timing of the arrival of farming at different locations [31], we estimated a narrow range of dates for when selection began (95% CI 6,256 to 8,683 years BP; mode = 7,441 years BP; see Figure 4A). Examples of plausible scenarios for the spread of the −13,910*T allele through time can be seen in Supplementary Videos S1, S2 and S3. These animations graphically represent the geographic frequency distribution of the −13,910*T allele in 10-generation time slices as taken from simulations that fitted best to data on modern −13,910*T allele frequency and timing of the arrival of farming.

#### Genetic contribution of the earliest LP dairying farmers to the modern European gene pool

**Figure 5:**
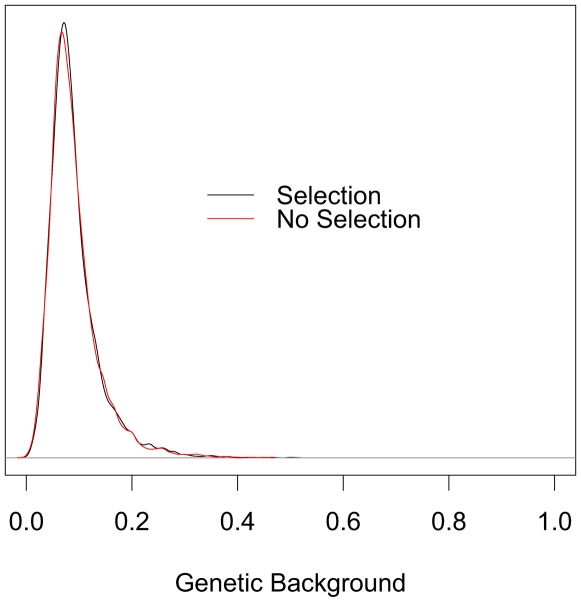
Contribution of people living in the deme of origin for LP/dairying co-evolution, and its 8 surrounding demes, to the modern European gene pool with and without selection on LP. Value distributions were taken from 5,000 simulations assuming selection (black line), and 5,000 simulations assuming no selection (red line). Simulation parameter values were sampled at random from the marginal posterior density estimates presented in Figure 1 and were identical for each set of 5,000 simulations, except that in the ‘no selection’ set the selection acting on the LP allele in dairyers parameter was set to zero.

Although not strictly a parameter of the model presented we have applied the ABC approach to estimate the genetic contribution of people living in the deme where LP-dairying gene-culture coevolution began, and its 8 surrounding demes, to the modern European gene-pool (95% CI 2.83 to 27.4%; mode = 7.47%; see Figure 4B). The genetic contribution will, to a large extent, be determined by the start location of LP-dairying gene-culture co-evolution. For example, if this process started in Anatolia or the Greek peninsula then we would expect the people living in that region to make a greater contribution to overall European ancestry than if it started in Northwest Europe. With respect to LP a more pertinent question is: Does the advent of LP-dairying coevolution increase the genetic contribution of people living in a particular region to the modern European gene pool? To investigate this we performed two extra sets of 5,000 simulations each by picking parameter values at random from the marginal posterior distributions obtained above. Each set of 5,000 simulations was run with identical sets of parameter value combinations except that in one set we fixed the level of selection acting on the LP allele to zero. We then compared the distributions of genetic contribution (of people living in and around the LP-dairying start deme to the modern European genepool) with and without selection acting. To our surprise the two distributions are nearly identical (see Figure 5).

#### Performance of model in explaining observed data

To explore the power of our model to explain the two data sets we have considered (13,910*T allele frequency at 12 European locations and farming arrival date at 11 European locations) we plotted the following for each data type and at each location considered: (1) the observed value, (2) the distribution of values from simulations accepted at the 0.5% tolerance level, and (3) the distribution of values from all simulations in which the 13,910*T allele arose and did not go extinct (see Supplementary Figures S1 and S2). Although it will necessarily be the case that the 0.5% closest points will be nearer to the observed summary statistics than those simulated from the prior, it is still possible that an observed value will be an outlier from the distribution of simulated points, possibly indicating poor fit of the model. However, as can be seen from Supplementary Figure S2, simulations accepted at the 0.5% tolerance level generate narrow ranges of expectations for the farming arrival date, in very good accordance with the observed (target) values. This can be taken to indicate that with our ABC-estimated parameter values, our model explains the farming arrival dates very well. When we consider the 13,910*T allele frequency at the 12 European locations for which we have data (Supplementary Figure S1) it is notable that the observed (target) values are within the 95% equal tail probability interval of expectations generated from simulations accepted at the 0.5% tolerance level. However, a number of the target values are somewhat offset from the expectation modes. In particular, it is notable that for northern European locations the observed frequency is lower than the mode of the expected values and the opposite is the case for southern European locations.

## Discussion

The simulation model we have employed here is relatively complex compared to related human demographic/evolutionary models reported [33]–[36]. The inclusion of a selected allele and three distinct but interbreeding cultural groups is necessary for the type of questions we are addressing. But the inclusion of four parameters related to sporadic migration activity, namely the proportion of individuals available for sporadic long-distance migration and the sporadic mobility of each of the 3 cultural groups (modeled separately as a Gaussian random walk process) both allows us to tackle the problem of migration overseas and adds, in our view, an extra level of realism to the model. However, as with any simulation model of population history, many simplifying assumptions have to be made and the extent to which these assumptions may lead to erroneous conclusions remains unknown. For example, we have not considered the ‘reverse-cause’ hypothesis [14], [17]–[19] – which proposes that dairying first arose in populations that were already LP – because both ancient DNA evidence [27] and data from lipid residues on pots [26] are inconsistent with this view. However, this does not mean that once LP-dairying gene-culture coevolution was established, conversion to the culture of dairying was more likely in high LP frequency populations. Such a process is captured in our model to an extent, in that ‘cultural’ conversion is determined by the frequency of the receiving cultural group (see equation 4), and LP is unlikely to rise to high frequencies anywhere without the presence of dairying. Nonetheless, a more explicit treatment of this process may lead to different conclusions. Some parameters, such as those relating to the effects of climate zone/elevation, and the logistic growth rate, are fixed based on realistic assumptions [37]–[39]. For those parameters that are allowed to vary within a range we note that an important shortcoming is that in any single simulation their value is constant over the 360-generation duration of the run. This may be a particular issue for selection acting on an LP allele in *F_d_* (see below). Since we identify ‘good’ simulations using their fit to only two data sets (arrival time of farming and LP allele frequency, both at a range of geographic locations) it is unsurprising that our analysis is relatively uninformative for some parameters. However, inclusion of these parameters does serve to reflect uncertainty in their values.

Estimates of the arrival dates for farming the 11 locations we consider here were calculated as local weighted averages of calibrated carbon-14 dates [31] from a Gaussian sampling region. We set the standard deviation of this region at the average nearest neighbour distance to ensure that most of the carbon-14 data was used. However, the geographic density of carbon-14 dates is highly uneven across Europe and so the number of such dates that are informative for farming arrival time at any of the 11 locations will vary. Also, there appears to be a considerable amount of noise in the dates for the first farmers. For example, the earliest carbon-14 date for farming in Ireland predates those for Great Britain, the Low Countries and Denmark. To test if these concerns had a major effect on our results we reanalysed our simulation date by setting the target farming arrival dates as those inferred by assuming a constant rate of spread of farming (estimated at 0.9 km/year [31]) and calculating the great circle distance from Anatolia to each sampling location. The results of this reanalysis were very similar to those presented above (see Supplementary Figures S3, S4, S5, and S6),

We are well aware that the spread of the Neolithic over Europe was not as constant as our model assumes. After the arrival of the Neolithic in the Balkans, there is a pause of approximately 800 years before it starts to spread to Central Europe, and there is another pause of 1,000 years before it spreads further into the northern German lowlands and other parts of the northern Europe. Clearly, the carbon-14 dates we have used to estimate the farming arrival times will not fully reflect the complex history of neolithisation in all parts of the continent.

The list of parameters for which the marginal posterior distributions are notably narrower than their corresponding prior ranges (selective advantage, intrademic gene flow, the sporadic migration distance of *F_d_* and *F_nd_*, and the geographic origin location of LP/dairying co-evolution) – which we interpret as those parameters for which our analysis is informative – is an unsurprising one since we would expect these parameters to have the greatest influence on the spread of an LP allele and farming in Europe. Likewise, it is unsurprising that the proportion available for sporadic migration and the sporadic mobility of (a) dairying farmers, and (b) non-dairying farmers are both strongly negatively correlated (Figure 2A and 2B) since we would expect these parameters to be confounded in influencing the arrival time of farmers at different locations.

The estimated selective advantage conferred by a LP allele (mode = 0.0953; 95% CI = 0.0518–0.159) is in good agreement with previous estimates for Europeans (0.014–0.15 [8]). However, it should be noted that (1) this estimate is for selection only in dairying farmers, who make up just under half of the population that we simulate, and (2) we assume that selection is constant over time. It is possible that selection favouring LP has in fact been episodic and possibly spatially structured in different climate zones [20], [40]–[44]. Episodic selection would be difficult to model without additional information on when those episodes were likely to have occurred. But we reason that constant selection strength is a more parsimonious assumption in the absence of evidence to the contrary. If, as modelled here, dairying farmers made up less than half of the European post-Neolithic population then we would expect the real continent-wide selection values for LP to average less that half of what we estimate here. Such a range of selection values are, however, still consistent with previous estimates based on haplotype decay [8].

Perhaps the most interesting result presented here is our estimation of the geographic and temporal origins of LP-dairying co-evolution. We find the highest posterior probabilities for a region between the central Balkans and central Europe (see Figure 3). At first sight such a location of origin may seem counter intuitive since it is far-removed from Northwest Europe, where the −13,910*T allele is found at highest frequency. However, previous simulations have shown that the geographic centroid of allele can be offset from its location of origin, particularly when it occurs on the wave front of a demographic expansion [29],[30]. The lactase-dairying coevolution origin region inferred here is consistent with a number of archaeologically attested patterns concerning the emergence and spread of dairying. Recent carbon isotope ratios from lipids extracted from archaeological sherds show the presence of milk fats in present-day western Turkey and connect these findings to an increased importance of cattle herding [26], [45]–[48]. In general, the spread of the Neolithic lifestyle from the Aegean to Central Europe goes hand in hand with the decline of the importance of sheep and goat and the rise in frequency of cattle bones in archaeological assemblages. While the Balkans at the beginning of the Neolithic still shows a variety of subsistence strategies [49], the middle Neolithic in SE-Europe and the earliest Neolithic in Central Europe after 7,500 BP show a clear preponderance of cattle. Benecke [50] gives the following averaged rates for the respective domestic species: cattle 55.2%, sheep and goat 32.6%, pig 12%. The proportion of cattle in Central Europe increases during the following centuries to an average of 73% and then stays (with a few exceptions) stable for most prehistoric periods of Middle and northern Europe. Thereby, cattle herding is in most cases connected with kill-of profiles indicative for dairying [22], [50]–[55]. Milk consumption and dairying have been proposed to be as early as the Pre-Pottery Neolithic B of the Near East and may even be a reason for domestication [56],[57]. Without doubt, it was a common cultural practice during all phases and regions of the European Neolithic, especially for goat and cattle. However, a fully developed dairying-based farming economy emerges first during the late Neolithic in Southeast Europe and the Middle Neolithic Cultures following the Linearbandkeramik (LBK) in Central Europe, and is connected mainly to cattle and partly also to goat (for the Rössen culture see [50],[55]). In the Mediterranean, milking of cattle occurs episodically [28] and sheep and goat remain the dominant domestics, as they were earlier in Anatolia and the Aegean. It is very likely that the goat and sheep, and to a lesser extent cattle, based economies of the Mediterranean used processed milk in the form of yoghurt, cheese and other milk-derived products instead of fresh milk. The nutritional and agricultural differences between southern Europe, the Mediterranean and central and northern Europe, as well as historic reports, point to this. For instance, the Romans used goat and sheep milk for the production of cheese, and cattle as a draught animal. In contrast the Germanic peoples and other inhabitants of central and northern Europe practised cattle dairying and drank fresh milk in significant amounts. Strabo reports in his Geography [58]: “Their [sc. “the men of Britain”] habits are in part like those of the Celti, but in part more simple and barbaric - so much so that, on account of their inexperience, some of them, although well supplied with milk, make no cheese; and they have no experience in gardening or other agricultural pursuits.”

Overall, by considering the results from our simulations and archaeological, archaeozoological, and archaeometric findings, it seems very plausible to connect the geographic origin of the spread of LP to the increasing emergence of a cattle-based dairying economy during the 6^th^ millennium BC. The geographic region of origin of the LBK – in modern day Northwest Hungary and Southwest Slovakia [59],[60] – certainly correlates well with our results (see Supplementary Figure S7). The date of origin of LP-dairying coevolution estimated here (mode = 7,441 years BP; 95% CI = 6,256 to 8,683 years BP; see Figure 4A and Supplementary Table S2) also fits well with dates for the early LBK in Central Europe (∼7,500 years BP) and its proposed main predecessor, the Starčevo culture of the northern Balkan Peninsula and south of Lake Balaton (8,100 to 7,500 years BP; [61]). However, as explained above, our date estimate is conditioned by farming arrival dates in the estimated LP-dairying coevolution origin region. As a result, our date and location estimates are not independently derived. Nonetheless, a role for LP-dairying coevolution in the later rapid spread of LBK culture – from its origins in the Carpathian Basin – into central and Northwest Europe would be consistent with the significantly higher sporadic migration distances we infer for of *F_d_* when compared to *F_nd_*. This is also consistent with the rapid dissemination of the LBK culture over a territory of 2,000 km width and approximately one million square kilometres within less than 500 years [62].

Contrary to our expectations, we did not find that the presence of a positively selected LP allele in early dairying groups increases the unlinked genetic contribution of people living in the region where LP-dairying coevolution started to the modern European gene pool, when using demographic parameter values estimated here. The main reason for this is likely to be the relatively high inferred rates of intra- and interdemic gene flow between dairying and non-dairying farmers and between neighbouring demes, respectively, leading to a rapid erosion of any demographic ‘hitchhiking’ of unlinked genomic regions. Additionally, we only track the genetic contribution of people living in and around the deme of LP/dairying coevolution from the inception of this process. Since it takes some time for the LP allele to rise to appreciable frequencies, any demographic ‘hitchhiking’ effect may become important only after the allele centroid has moved some distance away from its origin deme.

Another notable result was obtained when we compared the range of expected 13,910*T allele frequencies at different European locations – from simulations accepted at the 0.5% tolerance level – to those observed. While all observed values were within the 95% equal tail probability interval of the simulated values, many were somewhat offset from the modes. We interpret this as indicating that our model does not fully explain the distribution of the 13,910*T allele in Europe. One possible explanation for this is that migration activity – as modeled here by interdemic gene flow and sporadic unidirectional migration – has increased subsequent to the expansion of farming into the northwestern reaches of Europe. In this scenario the farming expansion phase, occurring 9,000 to 5,500 years BP, would be mainly responsible for generating the 13,910*T allele frequency cline in Europe but higher migration activity following this period would then have a homogenizing effect in LP allele frequencies. Intriguingly, a general pattern can be seen (Supplementary Figures 1) whereby observed frequencies are lower than expected in northern Europe and higher than expected in southern Europe. Such a pattern is the opposite of what we would expect if selection for LP was higher in northern latitudes through a greater requirement for dietary vitamin D and calcium because low-sunlight conditions reduce UV-mediated vitamin D production in the skin [20]. This frequently cited mechanism [9], [41], [42], [44], [63]–[65] was not included in our model and thus would seem to have negative explanatory power. Thus our simulations indicate that geographically and temporally homogeneous selection in combination with well-attested underlying demographic processes are sufficient to explain, indeed, to over-explain, the LP/latitude correlation in Europe. However, it should be noted that since we have not explicitly included a parameterised latitudinal effect on selection in our model, there may be scenarios where such an effect could also explain patterns of LP in Europe.

As inferred here, the spread of a LP allele in Europe was shaped not only by selection but also by underlying demographic processes; in this case the spread of farmers from the Balkans into the rest of Europe. We propose that this combination of factors could also explain the apparent homogeneity of LP-associated mutations in Europe. In Africa there are at least four known LP-associated alleles, including three that are likely to be of African origin [2],[4] as well as −13,910*T, which is likely to be of European origin [3],[12]. The greater apparent diversity of LP-associated mutations in Africa may reflect a greater genetic diversity in general, leading to the availability of more mutations upon which selection can act following the advent of dairying. However, we suggest that this diversity is the result of an ‘imposition’ of dairying culture on a pre-existing farming people, rather than the spread of dairying being tied to the spread of dairyers. Such a model would require the availability of a number of, albeit low-frequency, LP-causing mutations; either through a high mutation rate or a large number of potential LP-causing sites. It is therefore possible that, in the absence of the spread of dairying being linked to a major demographic expansion, high LP-allele diversity will also be found in the Indian subcontinent.

We accept that the model we have used does not accommodate all data (both genetic and archaeological) that is potentially informative on the coevolution of LP and dairying in Europe. Future improvements can be made by adding more ‘realism’ to the model and by increasing the number of data types that are used in the ABC analysis, leading to more integrative inference. The former should include both adding more fixed parameter information (such as the effects of past vegetation, climate variation and other geographic features on migration parameters and carrying capacities [66]–[68]) and estimating currently fixed parameters such as the ratio of dairying to non-dairying farmers. The latter could be achieved by writing the simulation model so that it generates expectations for other data types. For example, including the movement of domestic cattle could be used to generate expectations on patterns of ancient and modern cattle genetic diversity, for which considerable data is available [69]–[73]. Finally, it should be possible to extend the approach we have used to study the evolution of LP and dairying in other parts of the world.

We infer that the coevolution of European LP and dairying originated in a region between central Europe and the northern Balkans around 6,256 to 8,683 years BP. We propose the following scenario: after the arrival of the Neolithic in south-eastern Europe and the increasing importance of cattle herding and dairying, natural selection started to act on a few LP individuals of the early Neolithic cultures of the northern Balkans. After the initial slow increase of LP frequency in those populations and the onset of the Central European LBK culture around 7,500 BP, LP frequencies rose more rapidly in a gene-culture co-evolutionary process and on the wave front of a demographic expansion (see Supplementary Videos S1, S2 and S3), leading to the establishment of highly developed cattle- (and partly also goat-) based dairying economies during the Middle Neolithic of central Europe around 6,500 BP. A latitudinal effect on selection for LP, through an increased requirement for dietary vitamin D [20], is unnecessary to explain the high frequencies found in northern Europe.

## Material and Methods

Our simulation approach is motivated by a previous demic computer simulation study [33] and has features in common with more recent applications of this approach [34]–[36]. Geographic space is modelled as a series of rectangular demes arranged to approximate the European landmass (2375 land demes and 1511 sea demes). Each deme has attributes of elevation, area (which varies due to the curvature of Earth and is calculated accordingly for each individual deme), and a climate (Mediterranean, Temperate, or Cold/Desert – see Supplementary Figures S8 and S9). A maximum total population size is specified for each land deme taking into account its area, and assuming that lower elevation and mild Mediterranean climate results in a greater potential population size, while harsher conditions, such as high elevations and cold/desert climates, result in a smaller potential population size [38]. The ratio for the relative contribution coefficients of climate and elevation factors to the population size is fixed at 1∶4 in this study; meaning that elevation has a more dramatic effect than climate on population size. The maximum deme population size (carrying capacity, *K_deme_*, Supplementary Figure S10) is calculated by:
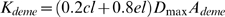
(1)where *cl* and *el* are the climatic and relative elevation factors, respectively; *cl* having values of 1 for Mediterranean, 2/3 for Temperate, and 1/3 for cold/desert climates [38] (see Supplementary Figure S9), and *el* being calculated as:
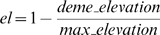
(1.1)


So *el* ranges between 0 at the highest elevation and 1 at sea level (see Supplementary Figure S8). *D_max_* is the maximum population density and is fixed at 5 individuals per km^2^ (i.e. in a sea level Mediterranean climate deme [39]), and *A_deme_* is the area of the deme in km^2^.

Each deme contains three distinct cultural groups: non-dairying farmers (F_nd_), dairying farmers (F_d_), and hunter-gatherers (HG). The ratios of ceiling population size for F_nd_, F_d_, and HG (as a proportion of the total maximum population size for the deme, *K_deme_*) are 50∶50∶1 respectively [37],[39]. Each cultural group in each deme is assigned a frequency for an allele that is subjected to genetic drift (modelled by intergenerational binomial sampling) and an allele at an unlinked locus that is not (as explained below). Initially the frequency of both ‘alleles’ is set at zero. The former represents a LP allele and is subject to selection of intensity *s*, only in the F_d_ group. The latter, here termed the GB (genetic background) ‘allele’, is used to track the general genetic ancestry component from the region where the LP allele is first found among dairying farmers. It will be used to infer the *expected* proportion of genes that originate from this region. The two alleles are assumed to be unlinked and are modelled separately. We treat *s* as an unknown but bounded parameter, and choose random values ranging from 0 to 0.2 in simulations [8].

The LP and GB ‘allele’ frequency dynamics are determined in each generation by five processes: (1) intrademic bidirectional geneflow between cultural groups; (2) bidirectional geneflow between demes (interdemic) within the same cultural groups; (3) sporadic unidirectional migration within the same cultural groups; (4) cultural diffusion (CD); and (5) selection operating on LP allele-carrying individuals within the F_d_ group. Hardy-Weinberg equilibrium within each cultural group within each deme is assumed. Population size increase for each cultural group in each deme is modelled by logistic growth, limited by the carrying capacity of each group within each deme. We fixed the growth rate to *r* = 1.3 per generation, a value estimated from data of world population growth rate over the last 10,000 years, excluding the post-Industrial Revolution population boom (US Census Bureau: www.census.gov). In addition, the F_d_ group is allowed to increase in size as a function of the selective advantage of the LP allele, *s*, by considering the number of LP individuals and the selective advantage to being a LP dairyer (see equation 7).

We define *intrademic bidirectional geneflow* as the exchange of individuals between different cultural groups within a deme (see Supplementary Figure S11). A proportion of individuals in each cultural group, *P_c_*, are deemed ‘available to change group’. The actual number of individuals that are exchanged between cultural groups *i* and *j*, *B_i↔j_*, is determined as follows:
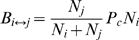
(2)Where *N_i_ and N_j_* are the total number of individuals belonging to each cultural group. We treat *P_c_* as an unknown but bounded parameter, and choose random values ranging from 0 to 0.2 in simulations [74],[75].

We define *interdemic bidirectional geneflow* as the exchange of individuals between the same cultural groups in neighbouring demes (see Supplementary Figure S11). A proportion of individuals in each cultural group, *P_d_*, are deemed ‘available to change deme’. The actual number exchanged is determined using the same formula as for *intrademic bidirectional geneflow* (equation 2), except we substitute *P_d_* for *P_c_*, and *N_i_ and N_j_* are the total number of individuals belonging to each cultural group in each neighbouring deme. In each generation, each cultural group in each deme undergoes *bidirectional geneflow* with one neighbouring deme, randomly chosen from the 8 possible.

We define *sporadic unidirectional migration* as the movement of some individuals in a particular cultural group and deme to the same cultural group in a different deme (see Supplementary Figure S11). A proportion of individuals in each cultural group, *P_s_*, are deemed ‘available to migrate’. The actual number of individuals that migrate, *N_mig_*, is dependent on the ‘pressure’ to leave the current deme and the availability of unoccupied carrying capacity in the destination deme (‘attractiveness’), and is determined as follows:
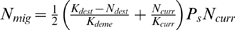
(3)Where *K_curr_* and *K_dest_* are the carrying capacities, and *N_curr_* and *N_dest_* are the number of people in the cultural group, in the current home and destination demes respectively. We treat *P_s_* as an unknown but bounded parameter, and choose random values ranging from 0 to 0.2 in simulations. The destination deme is chosen by a Gaussian random-walk process, which takes into account the mobility of the cultural group and the topography of the home deme. The Gaussian distribution is centred on the home deme; and its standard deviation is the product of the mobility of the cultural group, *M_i_*, and the relative mobility factor of the home deme, *M_curr_*. We treat *M_i_* as a separate unknown but bounded parameter for each of the three cultural groups, and choose random values ranging from 0 to 3 (demes) in simulations. *M_curr_* is determined for each deme by its elevation, allowing greater mobility at lower elevations [76],[77], with fixed values of 0.5 (demes) at mountainous terrain (above 1100 meters), 1.0 at lowlands (below 1100 meters), and 1.5 at coastal demes. The *sporadic unidirectional migration* function allows movement overseas, but whenever a sea deme is identified as a non-realistic destination deme the nearest neighbouring coastal deme is chosen instead. This feature, together with the attractiveness of low elevation land and the higher *M_curr_* value for coastal demes, creates the realistic tendency of a faster spread of farming along coastlines, consistent with archaeological data [78].

We define *Cultural Diffusion* (CD) as the spread of culture and technology by learning through exposure rather than by migration (see Supplementary Figure S11). In our simulations a proportion of individuals in each cultural group, *P_dif_*, are deemed ‘available to convert’ from one cultural group to another. The number of individual that convert from cultural group *i* to cultural group *j*, *N_i→j_*, is determined by this parameter and the proportion of the carrying capacity (*K*) of the home deme (deme *0*) and in the 8 neighbouring demes (demes *1* to *8*) that is taken up by cultural group *j*, as follows:
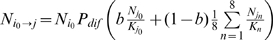
(4)where *b* is the relative influences of the home deme and the 8 neighbouring demes (fixed to 0.75). We treat *P_dif_* as an unknown but bounded parameter, and choose a random value ranging from 0 to 0.2 in each simulation. That value is then applied to ‘conversions’ between all 3 cultural groups.

The geographic location where LP/dairying gene-culture coevolution starts is chosen at random from all land demes. This LP mutation is initialized at a frequency of 0.1 in F_d_ when their population size reaches a critical size in the chosen start deme, set to a minimum of 20 individuals per deme in simulations. While we would expect any *de novo* mutation to always have an initial frequency of 1/2N, we also expect that it will have a high probability of extinction unless selection is very strong [79]. Indeed, in preliminary simulations this was observed (data not shown). Thus, for computational efficiency we condition on the LP mutation having already reached a frequency of 0.1 in F_d_ in the deme of origin. However, such a starting frequency means that little more than four LP alleles are initialized in simulations. Selection acting on the LP allele, *p*, increases its frequency in F_d_ only, as follows [80]:
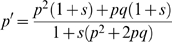
(5)where *s* is the selection coefficient for *p*, and *p′* is the new LP allele frequency. In addition, selection acting on the LP allele increases the number, *N*, in F_d_ as follows:
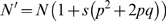
(6)where *N′* is the new number of F_d_ in a particular deme.

All simulations were run for 360 generations which, assuming a generation time of 25 years [81],[82], corresponds to the 9,000-year history of farming in Europe. We performed 200,000 simulations in total.

The genetic contribution of the population living in the region of origin of LP/dairying gene-culture coevolution to the overall European population is tracked over generations by calculating the GB ‘allele’ frequency over all demes in all 3 cultural groups. In the generation when the LP allele is initialized, all cultural groups in the origin deme and 8 neighbouring demes are assigned the unlinked GB ‘allele’ at a frequency of 1. The GB ‘allele’ is subjected to the same intra- and inter-deme geneflow and migration processes as described above, but is not subject to drift, as modelled by binomial sampling, or to selection. At the end of each simulation this GB allele is taken to represent the general genetic contribution of the population living in the region of origin of LP to the modern European population. The ancestry component of Europeans, at any generation, that originates from people living in the region of origin of the LP allele (*F_GB_*) is calculated as follows:
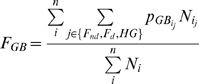
(7)where n is the number of land demes, *N_i_* is the total number of people in deme *i*, and *p_GBij_* and *N_ij_* are the frequency of the GB ‘allele’ and the population size in deme *i*/cultural group *j*, respectively.

To estimate parameters of interest we use an ABC approach, following [32]. By comparing summary statistics computed on each simulated dataset to those from the observed data, we are able to accept only those simulations with summary statistics sufficiently close to the target (i.e. the observed summary statistics) and reject the remainder. We then perform a weighted local-linear regression on these retained parameter sets, with weight determined by the “distance” between the simulation summary statistics and the target (all details below). This generates approximate marginal posterior probability distributions for each parameter of interest, from which we derive our modal point estimates. Our chosen summary statistics, **U**, are the frequencies of the −13,910*T allele at 12 different sample locations around Europe, the Near East and western Asia [7],[8]. In addition we include as summary statistics the times to arrival of farming at 11 of the same locations (the Anatolia location is excluded as the simulation model is initialized with this as the origin of the spread of farming into Europe). We recognize that these are not summary statistics *sensu stricto* but are parameters in the model for which we have independent estimates. However, the simulations, being stochastic, generate a distribution of arrival times, and we need to condition on those that are consistent with the known archaeological evidence. The most straightforward way to do this is to place a point prior on the arrival dates, and then condition on these using the ABC machinery, as if they are summary statistics. The point priors for the arrival dates of farming at 11 of the 12 sampling locations considered (Anatolia was set to 9,000 years as the simulations begin 360 generations ago in ‘an Anatolia’ populated by farmers) were calculated as follows: (1) The average nearest-neighbour distance (ANND) between each sampling location was calculated (557.13 km). (2) A 2-D Gaussian sampling region was constructed around each of the 11 sampling locations, of standard deviation = ANND/1.96 (this ensures that 95% of each Gaussian sampling region will be within the ANND). (3) A weighted average of all dates within 3 standard deviations of the sampling location was calculated using all calibrated carbon-14 earliest farming arrival dates from Pinhasi *et al.* [31], and weighting using the distance from the sampling location and the standard probability density function for a Gaussian distribution. Assuming a generation time of 25 years [81],[82] these observed dates are converted to generations from the start of the simulation, which was set at 9,000 years BP or 360 generations ago (see Table 1). We also include two Spearman's rank-order correlation coefficients, calculated separately for the 12 T-allele frequencies and the 11 times to arrival of farming, giving a total of 25 summary statistics. When calculating these statistics for the simulated data: we take LP frequencies in the final generation of the simulation at the 12 corresponding geographic locations; and the time to arrival of farming is defined as the simulation generation at which either *F_d_* or *F_nd_* reach 1% of their carrying capacity within each of the 11 corresponding location demes. All time to arrival of farming statistics are scaled to the interval [0,1] by dividing by the total number of simulated generations (360).

**Table 1:** −13,910*T allele frequencies, inferred farming start dates and geographic coordinates of 12 locations data used in ABC analysis.

Location	−13,910*T allele frequency	N individuals used to assess −13,910*T allele frequency	Reference for 13,910*T allele frequency	Great circle distance from central Anatolia (km)	Inferred farming arrival date in years BP ^1^ (generations after start of simulation)	Inferred farming arrival date in years BP ^2^ (generations after start of simulation)	Latitude	Longitude
Turkey	0.031	49	[7]	0	9000 (0)	9000 (0)	38.00	30.00
Greece	0.134	41	[7]	550	7932 (43)	8389 (24)	37.98	23.73
Tuscany	0.063	16	[8]	1699	7274 (69)	7112 (76)	43.77	11.25
Sardinia	0.071	56	[8]	1829	7371 (65)	6968 (81)	39.00	9.00
North Italy	0.357	28	[8]	1880	6992 (80)	6911 (84)	45.68	9.72
Scandinavia	0.815	360	[8]	2523	5833 (127)	6197 (112)	59.33	18.05
Germany	0.556	60	[7]	2309	6396 (104)	6434 (103)	53.55	10.00
France	0.431	58	[8]	2523	6552 (98)	6197 (112)	48.87	2.33
French Basque	0.667	48	[8]	2666	7078 (77)	6037 (119)	43.00	−1.00
Southern UK	0.734	64	[7]	2785	5954 (122)	5905 (124)	51.50	−0.12
Orkney	0.688	32	[8]	3325	5778 (129)	5306 (148)	58.95	−3.30
Ireland	0.954	65	[7]	3349	5807 (128)	5260 (150)	54.37	−7.63

Inferred arrival of farming dates were based on: ^1^ a weighted average of all calibrated carbon-14 earliest farming arrival dates from Pinhasi et al. [31] within 853 km of each sampling location, weighted using the distance from the sampling location and the standard probability density function for a Gaussian distribution of s.d. 285 km; and ^2^ by assuming a constant rate of spread of farming (estimated at 0.9 km/year [31]) and calculating the great circle distance from Anatolia to each sampling location. All inferred generations after the start of farming were calculated by assuming a generation time of 25 years [81],[82].

Parameters of interest, **φ**, are: the east-west and north-south coordinates of the location where the LP-allele first undergoes selection among *F_d_*; the generation at which this selection starts; the selective advantage of LP within the *F_d_* group, s; the proportion available for interdemic bidirectional geneflow, *P_d_*; the proportion available for intrademic bidirectional geneflow among cultural groups, *P_c_*; the rate of cultural diffusion, *P_dif_*; the proportion of people available for sporadic migration, *P_s_*; the mobility of each of the three cultural groups, *M_i_*; and the contribution of people living in the deme where LP-dairying gene-culture coevolution began and its 8 surrounding demes, *F_GB_*, to the modern European gene-pool. The uniform prior distributions for each parameter are given in Supplementary Tables S2 and S3.

Our full ABC algorithm is as follows: (1) choose the summary statistics **U** as outlined above and calculate their values, **u**, for the observed data (these are given in Table 1), (2) choose a tolerance level δ (as suggested we pre-define a proportion of the best fitting simulations, *P*_δ_, to accept and from this calculate an implicit tolerance level δ), (3) sample a parameter set φ_i_ from the pre-determined prior distribution of φ, (4) simulate forward under our model using parameter set φ_i_, (5) in the final generation of our simulation we calculate the summary statistics, **u_i_**, for this simulated data, (6) If ∥**u_i_**−**u**∥≤δ (where ∥.∥ is the Euclidean norm between the two vectors) we accept parameter set φ_i_, (7) steps 3 to 6 are repeated until we have a sufficient number of retained parameter sets, (8) A local-linear standard multiple regression is then performed to adjust the φ_i_, with each φ_i_ weighted according to the size of ∥**u_i_**−**u**∥ using the Epanechnikov kernel function K_δ_(t) (see [32] for details), (9) The resulting fitted parameter sets φ_i_* form a random sample from the approximate joint posterior distribution P(φ|**U** = **u**). All retained parameters – except for the two coordinate values and the generation at which the co-evolutionary process starts – were log transformed prior to the regression step, and subsequently back-transformed to produce the fitted parameter sets φ_i_*, as suggested by Beaumont *et al.* [32].

The simulation and ABC analysis procedures were written in the Python Programming Language (URL: http://www.python.org/) employing the numarray and Numpy array handling libraries. Maps and animations were generated using the Python library PyNGL. Post-ABC analysis data was processed and visualised using the statistical package ‘R’ (URL: http://www.R-project.org/).

## Supporting Information

Figure S1 Performance of model in explaining observed data on −13,910*T allele frequency at 12 locations throughout Europe. The observed point values are indicated by vertical red lines. The distributions of expected values from all simulations in which the 13,910*T allele arose and did not go extinct are indicated by black lines. The distributions of expected values from all simulations accepted at the 0.5% tolerance level in ABC analysis are indicated by green lines.
(0.62 MB EPS)

Figure S2 Performance of model in explaining observed data on the estimated time of arrival of farming at 11 locations throughout Europe. The observed point values are indicated by vertical red lines. The distributions of expected values from all simulations in which the 13,910*T allele arose and did not go extinct are indicated by black lines. The distributions of expected values from all simulations accepted at the 0.5% tolerance level in ABC analysis are indicated by green lines.
(0.60 MB EPS)

Figure S3 Approximate marginal posterior density estimates of demographic and evolutionary parameters. ABC was performed using regression adjustment and weighting, following acceptance at the 0.5% tolerance level. The upper and lower 2.5% of each distribution are shaded. These simulation results are equivalent to those presented in Figure 1 of the main text, but reanalysed after setting the target farming arrival dates as those inferred by assuming a constant rate of spread of farming (estimated at 0.9 km/year) and calculating the great circle distance from Anatolia to each sampling location.
(7.42 MB EPS)

Figure S4 Pairwise joint approximate posterior density estimates of demographic and evolutionary parameters showing high degrees of correlation (Spearman's R^2^>0.024). Points represent regression adjusted parameter values from simulations accepted at the 0.5% tolerance level. Shading was added using 2D kernel density estimation. These simulation results are equivalent to those presented in Figure 2 of the main text, but reanalysed after setting the target farming arrival dates as those inferred by assuming a constant rate of spread of farming (estimated at 0.9 km/year) and calculating the great circle distance from Anatolia to each sampling location.
(0.46 MB TIF)

Figure S5 Approximate posterior density of region of origin for LP/dairying co-evolution. Points represent regression-adjusted latitude and longitude coordinates from simulations accepted at the 0.5% tolerance level. Shading was added using 2D kernel density estimation. This result is equivalent to that presented in Figure 3 of the main text, but reanalysed after setting the target farming arrival dates as those inferred by assuming a constant rate of spread of farming (estimated at 0.9 km/year) and calculating the great circle distance from Anatolia to each sampling location.
(1.03 MB EPS)

Figure S6 Approximate marginal posterior density estimates of (a) the date of origin for LP/dairying co-evolution, and (b) the contribution of people living in the deme of origin for LP/dairying co-evolution, and its 8 surrounding demes, to the modern European gene pool. The upper and lower 2.5% of each distribution are shaded. These simulation results are equivalent to those presented in Figure 4 of the main text, but reanalysed after setting the target farming arrival dates as those inferred by assuming a constant rate of spread of farming (estimated at 0.9 km/year) and calculating the great circle distance from Anatolia to each sampling location.
(2.96 MB EPS)

Figure S7 Main regions of early (dark green) and late phase (light green) spread of the Linearbandkeramk culture from its origins in modern day northwest Hungary and southwest Slovakia.
(5.34 MB TIF)

Figure S8 Average deme elevation (scale bar in meters above sea level).
(0.47 MB EPS)

Figure S9 Deme climate zones: Mediterranean, Temperate, and Cold/Desert.
(0.39 MB EPS)

Figure S10 Carrying capacity (maximum number of people per deme; indicated by scale bar), calculated as a function of average deme elevation (Supplementary Figures S8), deme climate zones (Supplementary Figures S9) and curvature of the Earth (deme's area), as described in equation 1 of Materials and Methods.
(0.48 MB EPS)

Figure S11 Demographic processes: (a) Intrademic bidirectional geneflow - a single example deme is illustrated; bidirectional geneflow occurs between all cultural groups within the deme. The number of individuals exchanged between cultural groups *i* and *j*, *B_i⇔j_*, is calculated using equation 2 in the Material and Methods section; (b) Interdemic bidirectional geneflow - the central deme is illustrated as an example; the destination deme for geneflow within each cultural group is chosen at random from the 8 neighbours. The number of individuals exchanged between demes in each cultural group is calculated in an analogous way to intrademic bidirectional geneflow by modifying equation 2 (see Material and Methods section for details); (c) Sporadic unidirectional migration - only examples are illustrated as migrants potentially leave every populated deme. The migrants' destination deme is chosen by a Gaussian random walk process, centred on the home deme and with a standard deviation of the product of the cultural group mobility, *M_i_*, and the relative mobility factor of the home deme, *M_curr_* (see Material and Methods section for details); (d) Cultural diffusion - a single example deme for cultural group *i* is illustrated; the number of individuals in cultural group *i* converting to cultural group *j*, *N_i⇒j_*, is determined by the proportion of the carrying capacity (K) taken up by individuals of cultural group *j* in the home deme and the 8 neighbouring demes (see equation 4 in the Material and Methods section for details).
(0.06 MB DOC)

Table S1 Correlations among demographic and evolutionary parameters. Spearman's R^2^ (above diagonal) and p-values (below diagonal) are given for all pairwise joint posterior parameter distribution. Posterior distributions were estimated by ABC employing regression adjustment and weighting of simulations accepted at the 0.5% tolerance level. Parameter joint distributions are shown in Figure 2 (main article) for combination returning a Spearman's R^2^ value>0.024.
(0.06 MB DOC)

Table S2 Posterior estimates of demographic and evolutionary parameters (mean, mode and 95% credibility interval). Posterior distributions were by estimated by ABC employing regression adjustment and weighting of simulations accepted at the 0.5% tolerance level.
(0.06 MB DOC)

Table S3 Parameters of simulation model. ‘Flat’ indicates that a uniform prior was used.
(0.06 MB DOC)

Video S1 Supplementary Video S1 - Animation graphically representing the geographic frequency distribution of the −13,910*T allele at 10-generation time slices over the last 9000 years (assuming a generation time of 25 years), taken from simulations that best fitted data on modern −13,910*T allele frequency and timing of the arrival of farming in Europe.
(1.50 MB MPG)

Video S2 Supplementary Video S2 - Animation graphically representing the geographic frequency distribution of the −13,910*T allele at 10-generation time slices over the last 9000 years (assuming a generation time of 25 years), taken from simulations that best fitted data on modern −13,910*T allele frequency and timing of the arrival of farming in Europe.
(1.50 MB MPG)

Video S3 Supplementary Video S3 - Animation graphically representing the geographic frequency distribution of the −13,910*T allele at 10-generation time slices over the last 9000 years (assuming a generation time of 25 years), taken from simulations that best fitted data on modern −13,910*T allele frequency and timing of the arrival of farming in Europe.
(1.42 MB MPG)
