# Supplementary figures and images for: The Origins of Lactase Persistence in Europe
Source: PLoS Comput Biol. 2009 Aug 28;5(8):e1000491. doi: 10.1371/journal.pcbi.1000491 (PMC2722739; doi:10.1371/journal.pcbi.1000491)

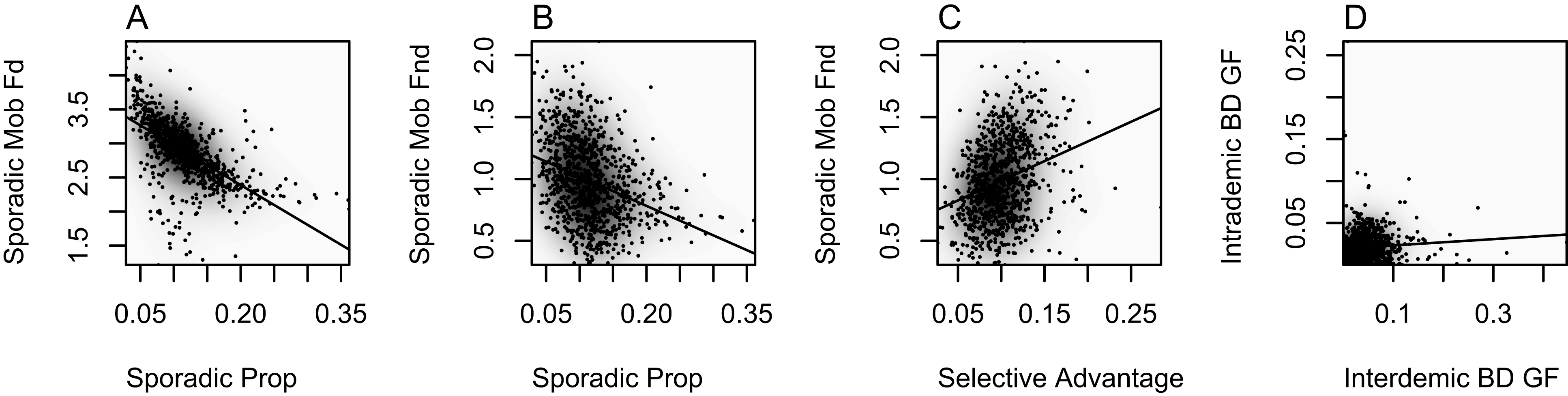

Supplement: Figure S4 — Pairwise joint approximate posterior density estimates of demographic and evolutionary parameters showing high degrees of correlation (Spearman's R2>0.024). Points represent regression adjusted parameter values from simulations accepted at the 0.5% tolerance level. Shading was added using 2D kernel density estimation. These simulation results are equivalent to those presented in Figure 2 of the main text, but reanalysed after setting the target farming arrival dates as those inferred by assuming a constant rate of spread of farming (estimated at 0.9 km/year) and calculating the great circle distance from Anatolia to each sampling location. (0.46 MB TIF) [file pcbi.1000491.s004.tif]

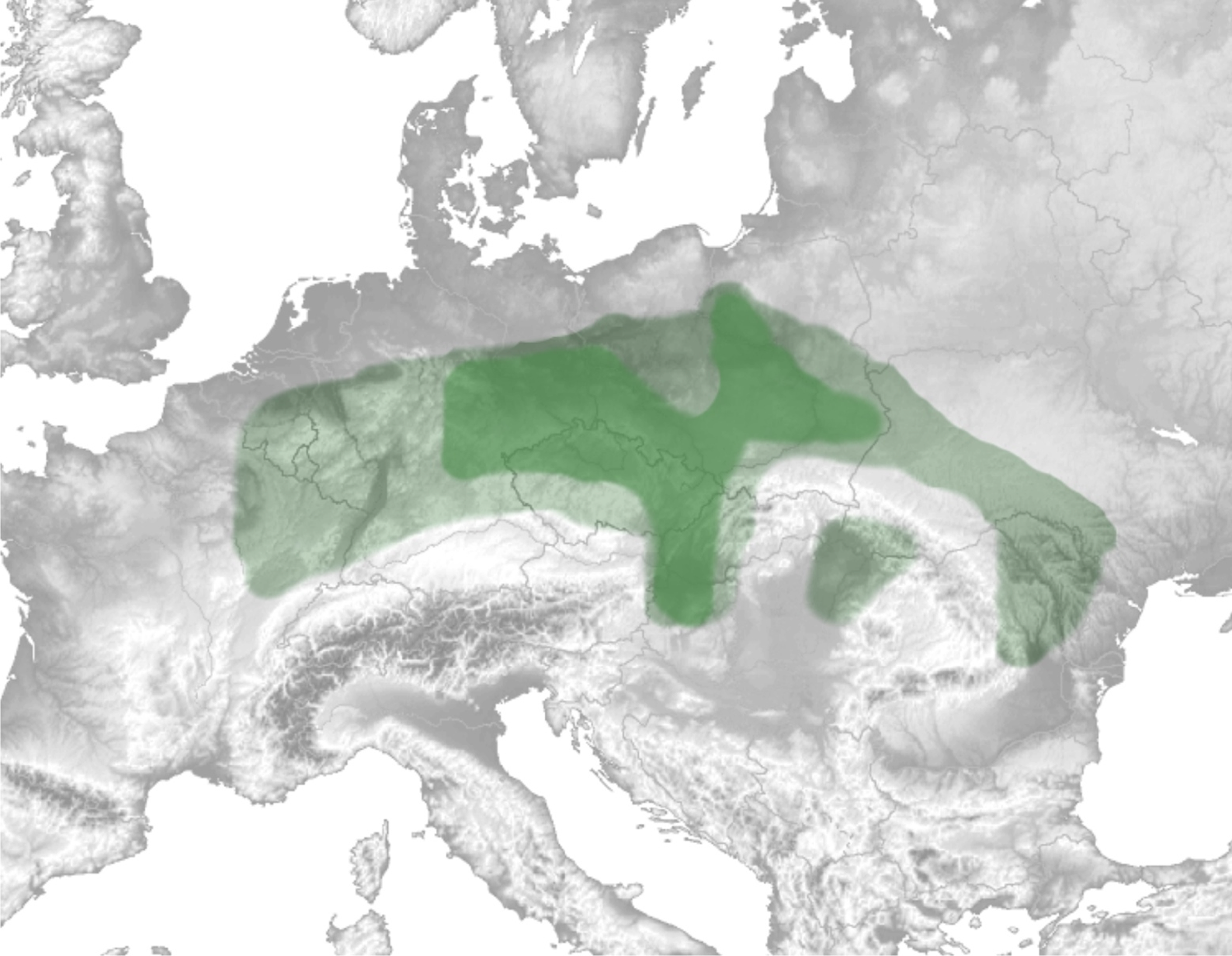

Supplement: Figure S7 — Main regions of early (dark green) and late phase (light green) spread of the Linearbandkeramk culture from its origins in modern day northwest Hungary and southwest Slovakia. (5.34 MB TIF) [file pcbi.1000491.s007.tif]
