# Supplementary material for: The Origins of Lactase Persistence in Europe
Source: PLoS Comput Biol. 2009 Aug 28;5(8):e1000491. doi: 10.1371/journal.pcbi.1000491 (PMC2722739; doi:10.1371/journal.pcbi.1000491)
Supplement: Figure S11 — Demographic processes: (a) Intrademic bidirectional geneflow - a single example deme is illustrated; bidirectional geneflow occurs between all cultural groups within the deme. The number of individuals exchanged between cultural groups i and j, Bi⇔j, is calculated using equation 2 in the Material and Methods section; (b) Interdemic bidirectional geneflow - the central deme is illustrated as an example; the destination deme for geneflow within each cultural group is chosen at random from the 8 neighbours. The number of individuals exchanged between demes in each cultural group is calculated in an analogous way to intrademic bidirectional geneflow by modifying equation 2 (see Material and Methods section for details); (c) Sporadic unidirectional migration - only examples are illustrated as migrants potentially leave every populated deme. The migrants' destination deme is chosen by a Gaussian random walk process, centred on the home deme and with a standard deviation of the product of the cultural group mobility, Mi, and the relative mobility factor of the home deme, Mcurr (see Material and Methods section for details); (d) Cultural diffusion - a single example deme for cultural group i is illustrated; the number of individuals in cultural group i converting to cultural group j, Ni⇒j, is determined by the proportion of the carrying capacity (K) taken up by individuals of cultural group j in the home deme and the 8 neighbouring demes (see equation 4 in the Material and Methods section for details). (0.06 MB DOC) [file pcbi.1000491.s011.doc]

**Supplementary Figure S11.**

**Demographic processes.**

**Supplementary Figure S11a**

**Intrademic bidirectional geneflow** – a single example deme is illustrated; bidirectional geneflow occurs between all cultural groups within the deme. The number of individuals exchanged between cultural groups *i* and *j*, *B_ij_*, is calculated using equation 2 in the **Material and Methods** section.

F_d_

F_nd_

HG

**Supplementary Figure S11b**

**Interdemic bidirectional geneflow** – the central deme is illustrated as an example; the destination deme for geneflow within each cultural group is chosen at random from the 8 neighbours. The number of individuals exchanged between demes in each cultural group is calculated in an analogous way to intrademic bidirectional geneflow by modifying equation 2 (see **Material and Methods** section for details).

F_d_

F_nd_

HG

**Supplementary Figure S11c**

**Sporadic unidirectional migration** – only examples are illustrated as migrants potentially leave every populated deme. The migrants’ destination deme is chosen by a Gaussian random walk process, centred on the home deme and with a standard deviation of the product of the cultural group mobility, *M_i_*, and the relative mobility factor of the home deme, *M_curr_* (see **Material and Methods** section for details).

F_d_

F_nd_

HG

**Supplementary Figure S11d**

**Cultural diffusion** – a single example deme for cultural group i is illustrated; the number of individuals in cultural group i converting to cultural group j, *N_ij_*, is determined by the proportion of the carrying capacity (K) taken up by individuals of cultural group j in the home deme and the 8 neighbouring demes (see equation 4 in the **Material and Methods** section for details).

Group i

Group j
