# Supplementary material for: The Origins of Lactase Persistence in Europe
Source: PLoS Comput Biol. 2009 Aug 28;5(8):e1000491. doi: 10.1371/journal.pcbi.1000491 (PMC2722739; doi:10.1371/journal.pcbi.1000491)
Supplement: Table S1 — Correlations among demographic and evolutionary parameters. Spearman's R2 (above diagonal) and p-values (below diagonal) are given for all pairwise joint posterior parameter distribution. Posterior distributions were estimated by ABC employing regression adjustment and weighting of simulations accepted at the 0.5% tolerance level. Parameter joint distributions are shown in Figure 2 (main article) for combination returning a Spearman's R2 value>0.024. (0.06 MB DOC) [file pcbi.1000491.s012.doc]

**Supplementary Table S1**

Correlations among demographic and evolutionary parameters. Spearman’s R^2^ (above diagonal) and p-values (below diagonal) are given for all pairwise joint posterior parameter distribution. Posterior distributions were estimated by ABC employing regression adjustment and weighting of simulations accepted at the 0.5% tolerance level [1]. Parameter joint distributions are shown in Figure 2 (main article) for combination returning a Spearman’s R^2^ value > 0.024.

|  | Sporadic Prop | Selective Advantage | Sporadic Mob F_d_ | Sporadic Mob F_nd_ | Interdemic BD GF | Intrademic BD GF | Cultural Diffusion | Sporadic Mob HG |
| --- | --- | --- | --- | --- | --- | --- | --- | --- |
| Sporadic Prop |  | 0.00680 | 0.458 | 0.118 | 1.69E-04 | 0.00229 | 0.00241 | 0.00710 |
| Selective Advantage | 0.00901 |  | 00727 | 0.0746 | 0.00222 | 0.0175 | 0.0111 | 5.96E-04 |
| Sporadic Mob F_d_ | 1.40E-135 | 0.00691 |  | 5.94E-04 | 0.00829 | 0.0137 | 0.0124 | 0.0255 |
| Sporadic Mob F_nd_ | 4.01E-29 | 1.27E-18 | 0.441 |  | 0.0208 | 1.90E-05 | 8.89E-05 | 0.00418 |
| Interdemic BD GF | 0.681 | 0.136 | 0.00390 | 4.52E-06 |  | 0.0239 | 0.00451 | 0.00197 |
| Intrademic BD GF | 0.130 | 2.59E-05 | 2.08E-04 | 0.890 | 8.53E-07 |  | 0.00580 | 4.18E-05 |
| Cultural Diffusion | 0.121 | 8.21E-04 | 4.14E-04 | 0.766 | 0.0334 | 0.0159 |  | 2.32E-04 |
| Sporadic Mob HG | 0.00760 | 0.440 | 3.70E-07 | 0.0406 | 0.160 | 0.838 | 0.630 |  |

1. Beaumont MA, Zhang W, Balding DJ (2002) Approximate Bayesian computation in population genetics. Genetics 162: 2025-2035.
