# Supplementary material for: The Origins of Lactase Persistence in Europe
Source: PLoS Comput Biol. 2009 Aug 28;5(8):e1000491. doi: 10.1371/journal.pcbi.1000491 (PMC2722739; doi:10.1371/journal.pcbi.1000491)
Supplement: Table S2 — Posterior estimates of demographic and evolutionary parameters (mean, mode and 95% credibility interval). Posterior distributions were by estimated by ABC employing regression adjustment and weighting of simulations accepted at the 0.5% tolerance level. (0.06 MB DOC) [file pcbi.1000491.s013.doc]

**Supplementary Table S2**

Posterior estimates of demographic and evolutionary parameters (mean, mode and 95% credibility interval). Posterior distributions were by estimated by ABC employing regression adjustment and weighting of simulations accepted at the 0.5% tolerance level [1].

| Parameter | Parameter symbol | Prior range | Units | Posterior 95% CI | Mode | Mean |
| --- | --- | --- | --- | --- | --- | --- |
| Interdemic BD GF | *P_d_* | 0 to 0.2 | Proportion | 0.00716 - 0.171 | 0.0440 | 0.0620 |
| Intrademic BD GF | *P_c_* | 0 to 0.2 | Proportion | 0.00206 - 0.0867 | 0.0153 | 0.0339 |
| Cultural Diffusion | *P_dif_* | 0 to 0.2 | Proportion | 0.00113 - 0.0847 | 0.0136 | 0.0321 |
| Selective Advantage | *s* | 0 to 0.2 | Proportion | 0.0518 - 0.159 | 0.0953 | 0.0957 |
| Proportion available for Sporadic migration | *P_s_* | 0 to 0.2 | Proportion | 0.0575 - 0.251 | 0.129 | 0.132 |
| Sporadic migration mobility HG | *M_HG_* | 0 to 3 | Demes | 0.333 - 2.17 | 1.16 | 1.20 |
| Sporadic migration mobility F_nd_ | *M_Fnd_* | 0 to 3 | Demes | 0.311 - 1.18 | 0.733 | 0.713 |
| Sporadic migration mobility F_d_ | *M_Fd_* | 0 to 3 | Demes | 2.15 - 3.93 | 3.12 | 3.08 |
| Time of Origin of Gene-Culture coevolution | Not a parameter *sensu stricto* | [0 to 9000] | Years | 6256 - 8683 | 7441 | 7553 |
| Genetic contribution to modern European genepool | Not a parameter *sensu stricto* | [0 to 100] | Percent | 2.83 - 27.4 | 7.47 | 11.1 |

1. Beaumont MA, Zhang W, Balding DJ (2002) Approximate Bayesian computation in population genetics. Genetics 162: 2025-2035.
