# Supplementary material for: The Origins of Lactase Persistence in Europe
Source: PLoS Comput Biol. 2009 Aug 28;5(8):e1000491. doi: 10.1371/journal.pcbi.1000491 (PMC2722739; doi:10.1371/journal.pcbi.1000491)
Supplement: Table S3 — Parameters of simulation model. ‘Flat’ indicates that a uniform prior was used. (0.06 MB DOC) [file pcbi.1000491.s014.doc]

**Supplementary Table S3**

Parameters of simulation model. ‘Flat’ indicates that a uniform prior was used.

| **Symbol** | **Fixed/ variable (F/V)** | **Value** | **Description** |
| --- | --- | --- | --- |
| *D_max_* | F | 5 | Maximum population density per per km^2^. |
| *cl* | F | cold=1/3, temperate= 2/3, med=1 | Climatic factor modifying carrying capacity. |
| *el* | F | 0 to 1, depending on elevation values as a proportion of max elevation. | Elevation factor modifying capacity. |
| *a* | F | 0.2 | Coefficient for relative contribution of climatic factor (a) and elevation factor (1-a) to deme carrying capacity. |
| *ratios* | F | 1:50:50 for HG, Fd, Fnd, respectively. | Ratios between the carrying capacities of the cultural groups, summing to deme's carrying capacity. |
| *r* | F | 1.3 | Logistic population growth rate. |
| *gen* | F | 360 | Number of generations in one simulation run. One generation = 25 years. |
| *M_curr_* | F | 1.5, 1, 0.5 for coastal, lowland, and mountains, respectively. | Topography factor modifying sporadic migration distance. Mountains defined as elevation>1100m |
| *b* | F | 0.75 | Cultural diffusion coefficient for relative contribution of local population density and 1-b for surrounding demes' population density. |
| *s* | V | 0 to 0.2 (flat) | Selective advantage. Affects gene frequencies and population growth. |
| *P_c_* | V | 0 to 0.2 (flat) | Proportion of people available to move to another cultural group within a deme (bidirectional). |
| *P_d_* | V | 0 to 0.2 (flat) | Proportion of people available to move to the same cultural group in a neighbouring deme (bidirectional). |
| *P_s_* | V | 0 to 0.2 (flat) | Proportion of people available for sporadic migration. |
| *M_Fnd_* | V | 0 to 3 (flat) | Sporadic migration mobility of non-dairying farmers (s.d. of the Gaussian random walk distribution given by the product of this value, *M_i_*, and the relative mobility factor of the home deme, *M_curr_*). |
| *M_Fd_* | V | 0 to 3 (flat) | Sporadic migration mobility of dairying farmers. |
| *M_HG_* | V | 0 to 3 (flat) | Sporadic migration mobility of hunter-gatherers. |
| *P_dif_* | V | 0 to 0.2 (flat) | Maximum proportion of people available for converting into another cultural group. |
| *location* | V | Any land deme | Start location coordinates for LP-dairying coevolution. |
